# Genetic Deletion of SEPT7 Reveals a Cell Type-Specific Role of Septins in Microtubule Destabilization for the Completion of Cytokinesis

**DOI:** 10.1371/journal.pgen.1004558

**Published:** 2014-08-14

**Authors:** Manoj B. Menon, Akihiro Sawada, Anuhar Chaturvedi, Pooja Mishra, Karin Schuster-Gossler, Melanie Galla, Axel Schambach, Achim Gossler, Reinhold Förster, Michael Heuser, Alexey Kotlyarov, Makoto Kinoshita, Matthias Gaestel

**Affiliations:** 1 Institute of Physiological Chemistry, Hannover Medical School, Hannover, Germany; 2 Department of Molecular Biology, Division of Biological Science, Nagoya University Graduate School of Science, Furo, Chikusa, Nagoya, Japan; 3 Department of Hematology, Hemostasis, Oncology and Stem Cell Transplantation, Hannover Medical School, Hannover, Germany; 4 Institute of Immunology, Hannover Medical School, Hannover, Germany; 5 Institute of Molecular Biology, Hannover Medical School, Hannover, Germany; 6 Institute of Experimental Hematology, Hannover Medical School, Hannover, Germany; 7 Division of Hematology/Oncology, Boston Children's Hospital, Harvard Medical School, Boston, Massachusetts, United States of America; Washington University School of Medicine, United States of America

## Abstract

Cytokinesis terminates mitosis, resulting in separation of the two sister cells. Septins, a conserved family of GTP-binding cytoskeletal proteins, are an absolute requirement for cytokinesis in budding yeast. We demonstrate that septin-dependence of mammalian cytokinesis differs greatly between cell types: genetic loss of the pivotal septin subunit SEPT7 *in vivo* reveals that septins are indispensable for cytokinesis in fibroblasts, but expendable in cells of the hematopoietic system. SEPT7-deficient mouse embryos fail to gastrulate, and septin-deficient fibroblasts exhibit pleiotropic defects in the major cytokinetic machinery, including hyperacetylation/stabilization of microtubules and stalled midbody abscission, leading to constitutive multinucleation. We identified the microtubule depolymerizing protein stathmin as a key molecule aiding in septin-independent cytokinesis, demonstrated that stathmin supplementation is sufficient to override cytokinesis failure in SEPT7-null fibroblasts, and that knockdown of stathmin makes proliferation of a hematopoietic cell line sensitive to the septin inhibitor forchlorfenuron. Identification of septin-independent cytokinesis in the hematopoietic system could serve as a key to identify solid tumor-specific molecular targets for inhibition of cell proliferation.

## Author Summary

Cytokinesis is the finalizing step of the complex scenario of mitosis, leading to separation of two sister cells. The cellular mechanism of cytokinesis in eukaryotes differs at least between yeasts, plants and animals. So far, it is also not clear whether all mammalian cells follow the same mechanistic rules of cytokinesis. Here, we demonstrate that, depending on the mammalian cell type, two different pathways could result in completion of cytokinesis, a septin-dependent pathway and a distinct mechanism, which does not require septins prevalent in the hematopoietic system. Using multiple conditional knockouts, we demonstrate this cell type specificity *in vitro* and *in vivo*, and present evidence for the involvement of cell-type specific alteration of the microtubule cytoskeleton. Our data, together with the previously available septin knockdown data in cancer cell lines, suggest septins as plausible antitumor targets with high therapeutic index due to lack of off-target effects on hematopoiesis.

## Introduction

Cytokinesis as final step of cell division is essential for cell proliferation, but there is a considerable degree of diversity in its underlying mechanisms among eukaryotes. Even within one organism, such as the amoeba *Dictyostelium discoideum*, cytokinesis may proceed by different mechanisms for cells growing in suspension or in an attachment-dependent manner. This has been impressively demonstrated for the myosin II-deletion mutant of *D. discoideum*, which could not further complete cytokinesis in suspension but successfully proliferates when attached to surfaces [1]. Hence, it could be speculated that other cells also confine different molecular requirements for attachment-dependent and -independent cytokinesis, although there is little molecular proof for this idea in mammalian cells. Recent support for this idea comes from the observation that in lymphocytes the hematopoietic linage-specific Rho-GAP ARHGAP19 is essential for cytoskeleton remodeling resulting in cell division [2] while in most other cells M-phase GAP (MP-GAP) is the major factor restraining RhoA during cell division [3].

Septins, a conserved family of polymerizing GTP-binding proteins regarded as the forth component of the cytoskeleton [4], organize a ring that serves as a submembranous scaffold and diffusion barrier for various molecules, which is an absolute requirement for cytokinesis in budding yeast [5], [6]. In metazoans, septins associate with the mitotic spindle, contractile ring, intercellular bridge and midbody at varying degrees [7], [8]. For example, anillin-dependent recruitment of septins to the intercellular bridge is required for constriction site formation and ingression in HeLa cells [9], maturation of the midbody ring in *Drosophila melanogaster* requires septin-dependent removal of anillin via its C-terminal PH-domain [10], and septins are required for the release of midbody and midbody ring into daughter cells during the subsequent cell division in *Caenorhabditis elegans* [11]. Perturbation or depletion of one of the major septin subunits, such as the pivotal subunit SEPT7 [12], [13], affects multiple steps in mitosis [4], [14]. *In vitro* studies with mammalian cell lines have revealed pleiotropic defects in mitotic spindle organization and chromosome alignment [15], cleavage furrow ingression [16], and midbody abscission [17], [18]. Intriguingly, however, depletion of each septin subunit in adherent cells by RNAi abolishes cytokinesis only at low penetrance (<25%) [15], [18], [19]. Further, mitosis is completely unaffected in T lymphocytes depleted for the pivotal subunit SEPT7 [20]. To explore the molecular mechanism underlying the relative and cell-type specific requirement of septins in physiological systems we manipulated the *Sept7* gene in mice and analyzed cytokinesis of cells with deleted *Sept7*.

## Results

### Deletion of *Sept7* causes embryonic lethality

**Figure 1:**
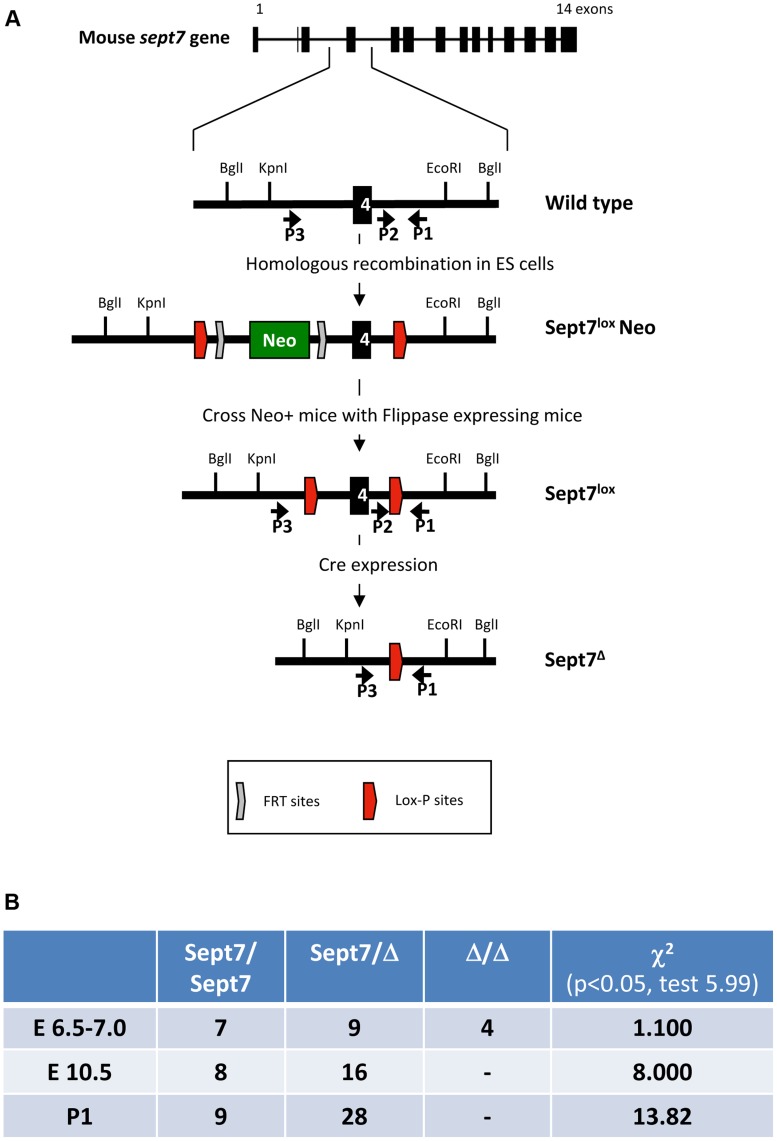
Generation of *Sept7* floxed mice and characterization of embryonic lethality of the *Sept7* knockout. **A,** Strategy for conditional targeting of *Sept7*. A neomycin cassette with flanking FRT and lox-P sites was incorporated by homologous recombination in ES cells. The resulting mice were crossed with Flippase expressing mice to remove the neomycin cassette retaining the lox-P flanked (floxed) exon 4. Cre expression leads to excision of exon4 and a downstream frame shift. **B,** Analysis of embryonic lethality in *Sept7* knockout mice. Analysis of progeny by genotyping at postnatal day 1 (P1) and embryos at E6.5–7 and E10.5, shows embryonic lethality between E7.5 and E10.5. Significance of the χ^2^ test is given where a difference to Mendelian distribution is indicated by values greater than 5.99 (p = 0.05).

We floxed *Sept7* gene (exon 4, encoding the GTP-binding P-loop) in the mouse genome using the Cre-loxP system (Figure 1A). The *Sept7* ^flox^ allele was converted to *Sept7* ^−^ (null) allele by using oocyte-specific expression of Cre-recombinase (ZP3-Cre). *Sept7* ^−/−^ (KO) embryos were found *in utero* up to embryonic day 6.5 (E6.5)-E7.0, but not after E10.5, indicating early embryonic lethality (Figure 1B). As the genetic loss of SEPT9 or SEPT11 causes embryonic death by E10 [21] and E13 [22] respectively, SEPT7 appears no less vital than these major subunits. These data obviously indicate that septins are dispensable for the majority of cells to execute mitosis in early mouse embryo.

### SEPT7-deficient fibroblasts display incomplete cytokinesis and constitutive multinucleation

**Figure 2:**
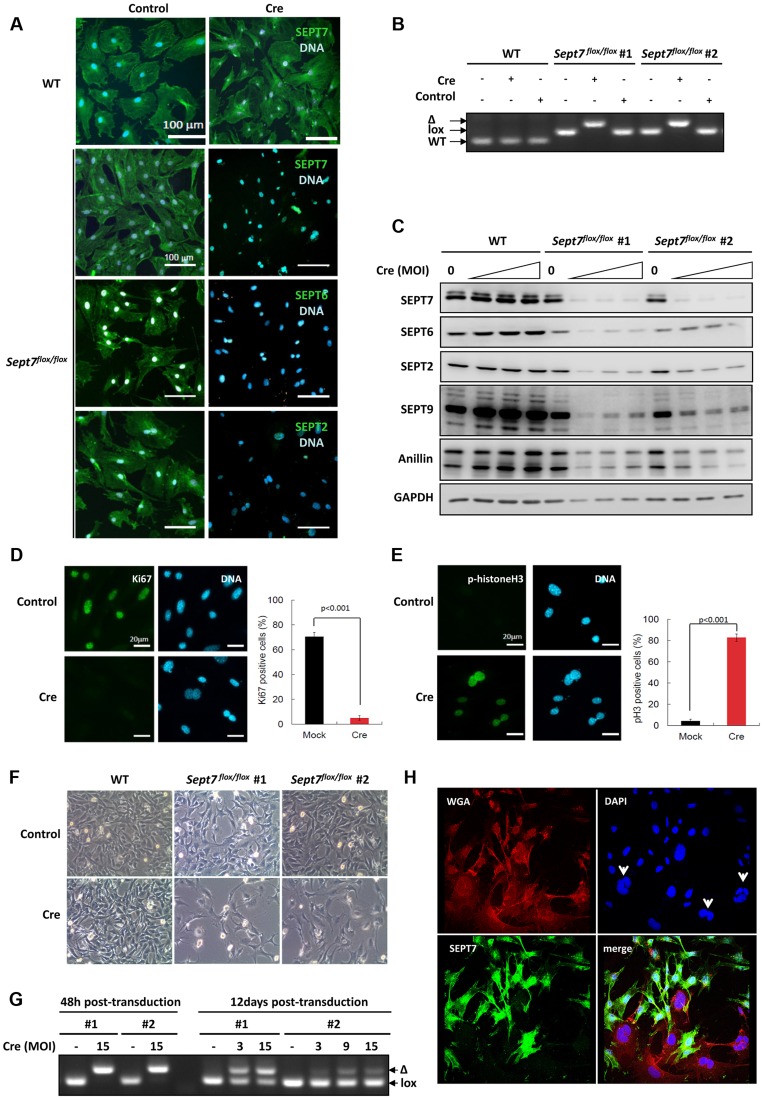
Cre-induced deletion of SEPT7 in fibroblasts leads to proliferation block and obligate multinucleation. **A**, Adenoviral Cre-expression in *Sept7* ^flox/flox^ primary mouse embryonic fibroblasts (MEFs) [26] leads to deletion of SEPT7 and, as judged by immunofluorescence, to a strong reduction of expression of the other core-septins SEPT6/2. **B**, Retroviral Cre-transduction of *Sept7* ^flox/flox^ immortalized mouse tail fibroblasts causes complete deletion of the *Sept7* gene in two different experiments (#1, #2- independently immortalized lines) (control: non-integrating control virus). Genotyping by PCR as indicated in Supporting Figure S1. **C**, Western blot detection of multiplicity of infection (MOI)-dependent reduced SEPT7 levels in *Sept7* ^flox/flox^ immortalized mouse tail fibroblasts. Expression of SEPT6/2/9 and anillin is significantly reduced. **D,E,** Adenoviral Cre-expression in primary *Sept7* ^flox/flox^ MEFs decreases immunofluorescence detection of the proliferation marker Ki67 (D) and increases histone H3 phosphorylation (E). The percentage of positive cells is indicated. **F,** SEPT7-depleted *Sept7* ^flox/flox^ immortalized mouse tail fibroblasts fail to proliferate (3 days post transduction). **G,** Retroviral Cre-transduced SEPT7-depleted cells are overgrown by non-depleted cells, as indicated by the relative increase in the floxed allele after 12 days in culture. **H,** Immunofluorescence analysis showing obligatory multi-nucleation of *Sept7*-KO tail fibroblasts after 11 days post transduction. DAPI is used for nuclear staining and the WGA as counter stain. Arrowheads indicate multi-nucleated, SEPT7-negative cells.

To probe the impact of the genetic loss of SEPT7 on mitosis *in vitro*, we prepared primary fibroblasts (MEFs) and SV40-large T-immortalized tail fibroblasts (TFs) from *Sept7* ^flox/flox^ mice. Cre-transduction via adeno- or retroviral vectors caused significant reduction of SEPT7 and collateral depletion of SEPT2, SEPT6, and SEPT9 (Figure 2A,2C) [20], [23], following the deletion of the exon 4 (Figure 2B). Of note, a septin-binding contractile ring protein anillin was also reduced (Figure 2C) down to 26%–88% depending on the cell line and multiplicity of infection (cf. Figure S1). Consequently, *Sept7* ^−/−^ MEFs arrested at G2/M in the cell cycle, as was indicated by the absence of a proliferation marker Ki67, remarkable phosphorylation of histone H3 and decreased overall proliferation (Figure 2D–2F) without increased apoptosis (Figure S2). The incomplete efficiency in infection and/or recombination (Figure 2G) caused a *Sept7* ^flox/flox^/*Sept7* ^−/−^ mosaic culture and a heterogeneity in SEPT7 level after 12 days, which demonstrated that cells without SEPT7 expression were almost exclusively multinucleated and significantly larger than the neighboring mononucleated cells with residual SEPT7 (Figure 2H and Figure S3A, S3D). In detail, of 223 SEPT7-positive cells analyzed by imaging, 222 cells (99,55%) were mono-nucleated. Of 56 SEPT7-negative cells, 54 cells (96,4%) were bi- (38 cells, 67,8%) or multinucleated (16 cells, 28,6%).

### Impaired cytokinesis and stalled midbody abscission in SEPT7-deficient fibroblasts

**Figure 3:**
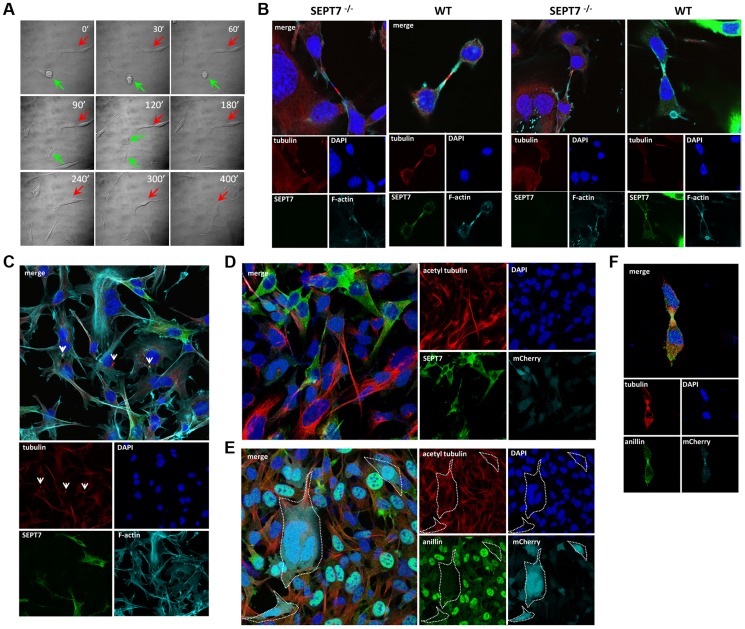
Defective cytokinesis and unresolved midbody in SEPT7-deficient fibroblasts. **A,** Time lapse differential interference contrast (DIC) microscopy of Cre-transduced floxed *Sept7* tail fibroblasts. The green arrow indicates a SEPT7-positive, normal dividing cell (about 70% of cells). The process of cell division is completed in less than 120 min and two daughter cells appear. The red arrow indicates a SEPT7-negative cell which could not complete cytokinesis after nuclear division (about 30% of cells). Even after 400 min the daughter cells do not separate and the cell becomes multi-nucleated. **B**, Localization of microtubules (α-tubulin) and microfilaments (phalloidin) by immunofluorescence in the midbody zone of dividing fibroblasts in the absence (SEPT7 −/−) or presence of SEPT7 (WT). Two different cells each were analyzed. Arrowheads indicate the midbodies. **C**, Detection of unresolved midbody tubulin bundles (indicated by arrowheads) in multinucleated SEPT7-deficient cells. **D,E**, Immunofluorescence of tail fibroblasts transduced with pRbid-Cre-mCherry. **D**, About two-fold increased acetyl-tubulin detection and **E**, about 2–3-fold decreased nuclear anillin staining in mCherry-Cre-positive, SEPT7-negative cells. **F**, Intact recruitment of the remaining anillin to the midbody zone in mCherry-positive, SEPT7-negative cells.

Time-lapse observation of the same population identified two subsets; one completed cytokinesis normally within 70–130 min (about 70% of cells), while another could not complete cytokinesis within 130 min, displaying stalled cytokinesis yielding binucleated cells after unsuccessful severing of the intercellular bridge (about 30% of cells) (Figure 3A, Figure S4 and video S1). Immunofluorescence analysis of the intercellular bridges and midbodies did not show obvious disorganization of α-tubulin and F-actin in the absence of SEPT7 (Figure 3B and Figure S3A, S3B, S3D). Improper segregation of chromosomes can lead to the formation of chromatin-bridges associated with a delay in abscission and multinucleation [24]. Analysis of the arrested midbody structures in the *Sept7* ^−/−^ revealed absence of persistent chromatin bridges as shown by LAP2 staining (Figure S5). However, *Sept7* ^−/−^ cells were often accompanied by unresolved α-tubulin aggregates (arrowheads in Figure 3C) and about two-fold hyperacetylation of α-tubulin (Figure 3D and Figure S3C, S3E). These data indicate hyperstabilization of microtubules in *Sept7* ^−/−^ cells, as has been observed in interphase HeLa cells [25] and postmitotic primary neurons [26]. Anillin, a contractile ring organizer which interacts with actomyosin and septins, was reduced in interphase nuclei of *Sept7^−/−^* cells (Figure 3E, cf. Figure 2C and Figure S1). However, SEPT7 was dispensable for the targeting of anillin to the cleavage furrow (Figure 3F). Thus, genetic loss of SEPT7 in fibroblasts appeared to affect mitotic spindle and midbody rather than the contractile ring.

### SEPT7 is dispensable for the cytokinesis of myeloid and lymphoid cells

**Figure 4:**
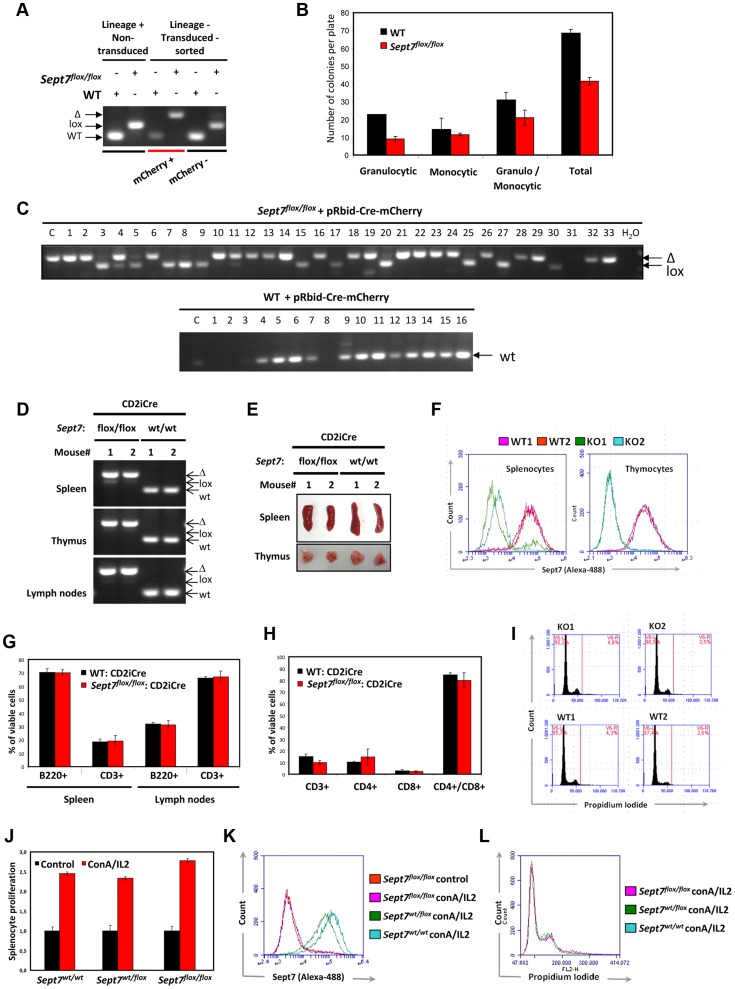
Intact cytokinesis, development and proliferation of myeloid and lymphoid SEPT7-deficient cells. **A–C,** Analysis of bone marrow-derived myeloid cells in a colony formation assay. **A,** Genotype analysis of lineage-positive bone marrow cells from WT and *Sept7* ^flox/flox^ mice and lineage negative mCherry Cre (mCherry +) – and control mCherry (mCherry −) -transduced and sorted cells. **B,** mCherry-positive Cre-transduced cells were seeded for myeloid colony formation assay in the presence of IL3/IL6/SCF. After 14 days the number of colonies per plate was counted, their morphology evaluated and the mean and SD of 2 plates are given. **C**, The macroscopic colonies were genotyped by PCR for detection of the *Sept7* deletion. Lane C denotes the analysis of the cells seeded for the assay. **D–H**, Analysis of the lymphoid cell-specific deletion of SEPT7 in the CD2-iCre::*Sept7* ^flox/flox^ cross. **D**, Complete deletion of *Sept7 exon4* in spleen, thymus and lymph nodes of CD2-iCre::*Sept7* ^flox/flox^ mice (two mice of each genotype #1, #2 were analyzed). **E**, Representative images of spleen and thymus of CD2-iCre::*Sept7* ^wt/wt^ and CD2-iCre::*Sept7* ^flox/flox^ mice. **F**, FACS analysis of SEPT7 expression in splenocytes and thymocytes of CD2-iCre::*Sept7* ^wt/wt^ (WT) and CD2-iCre::*Sept7* ^flox/flox^ (KO) mice. **G,H**, Cell surface marker analysis of spleen and lymph nodes (**G**) and thymus (**H**) of CD2-iCre::*Sept7* ^wt/wt^ and CD2-iCre::*Sept7* ^flox/flox^ mice. **I**, Nuclear staining displays no increased multinucleation of CD2-iCre::*Sept7* ^flox/flox^ (KO) thymocytes. **J–L**, *In vitro* splenocyte proliferation assay. **J**, ConA/IL-2-induced proliferation of CD2-iCre mice with the different *Sept7* genotypes. Viable cells present 24 h after induction were determined using WST1 assay and compared to non-induced control cells (mean and SD of triplicate wells are given). **K**,**L**, FACS analysis of SEPT7 expression (K) and multinucleation (L) of the splenocytes analysed in J.

Next, we examined the aforementioned presumed dispensability of SEPT7 in non-adherent cell lineages. We introduced a bidirectional γ-retroviral mCherry-Cre construct [27] (Figure S6A, 6b) into *Sept7* ^flox/flox^ bone marrow cells, which successfully induced recombination (Figure 4A). An interleukin (IL)-3/IL-6/SCF-dependent myeloid colony formation assay (Figure S6C) revealed that each subpopulation of the *Sept7* ^−/−^ leukocytes exhibited subnormal but sufficient proliferative activity *in vitro* (Figure 4B). Given that most of these *Sept7* ^−/−^ cells (Figure 4C) had undergone more than 10 replication cycles, SEPT7 protein carried over from the original *Sept7* ^flox/flox^ cell had been eliminated. These data indicate that the resistance to the loss of SEPT7 in mitosis is a common trait of the myeloid lineage.

To corroborate the dispensability of SEPT7 in myeloid cell mitosis *in vivo*, we generated lymphocyte-specific *Sept7* ^−/−^ mice, by intercrossing *Sept7* ^flox/flox^ and CD2-iCre lines [28]. We detected efficient recombination in the bone marrow (Figure S7A), spleen, thymus, and lymph nodes (Figure 4D) with recognizable volume loss in the spleen and thymus (Figure 4E). Flow cytometric analysis demonstrated complete loss of SEPT7 in cells collected from thymus, while those from spleen contained a minor population that fully expressed SEPT7 (Figure 4F). Viability of lymphocytes from spleen, peripheral lymph nodes (Figure 4G), thymus (Figure 4H), bone marrow (Figure S7B) and a number of peripheral blood cells (Figure S8) showed no differences with or without *Sept7*. Although SEPT7/6/2/9 had been depleted from *Sept7* ^−/−^ thymocytes (Figure S9), flow cytometric DNA content analysis did not detect any multinucleated population (Figure 4I). Intriguingly, as opposed to fibroblasts, HeLa cells [25] and neurons [26], thymocytes did not exhibit microtubule hyperacetylation after septin depletion (Figure S10). *Sept7* ^−/−^ splenocytes proliferated normally *in vitro* in response to concanavalin A and IL-2 (Figure 4J, 4K), without forming multinucleated cells (Figure 4L). Taken together, we conclude that *Sept7* is dispensable in the proliferation and maturation of B- and T-lymphocytes *in vivo*, and in the proliferation of splenocytes and myeloid progenitors *in vitro*.

### Elevated levels of stathmin enable SEPT7-deficient cells to complete cytokinesis

**Figure 5:**
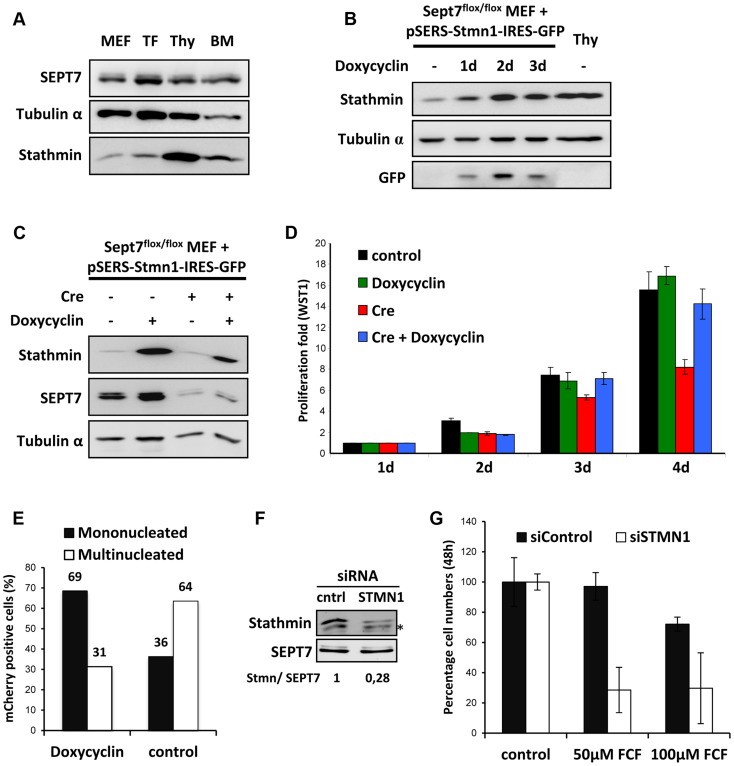
Stathmin expression levels correlate with SEPT7 dependence on cytokinesis. **A,** Lysates prepared from mouse embryonic fibroblasts (MEF), tail fibroblasts (TF), thymocytes (Thy) and bone marrow cells (BM) were probed with indicated antibodies. **B,** *Sept7* ^flox/flox^ MEFs transduced with inducible stathmin expression construct were treated with 1 µg/ml doxycycline, to induce stathmin expression similar to thymocytes (Thy). **C,** Cre-induced SEPT7 depletion in *Sept7* ^flox/flox^ cells in the presence and absence of doxycycline induced stathmin expression (5 days). **D,** WST-1 assay showing the rescue of Cre-transduction-induced proliferation defect in fibroblasts by doxycycline-induced stathmin expression. **E,** Effect of doxycycline induced stathmin expression on Rbid-Cre induced multinucleation analyzed and quantified from fluorescent images as indicated in Figure S12. **F, G**, Knockdown of stathmin in Jurkat cells. **F**, Stathmin expression was reduced to about 28% by knockdown. **G**, Stathmin knockdown renders Jurkat cell proliferation sensitive to the septin inhibitor forchlorfenuron (FCF) applied at two different concentrations (50 and 100 µM).

In our search for the factor enabling diverse hematopoietic cell lineages to go through the cell cycle without SEPT7, we compared the proteome between the fibroblasts and myeloid cells. From a number of candidate proteins we focused our studies on stathmin (STMN1) because of its specific abundance in the blood cell lineages (Figure 5A) and biochemical activity. The stathmin family is known to facilitate microtubule depolymerization by sequestering α/β-tubulin heterodimers [29], [30]. We hypothesized that the scarcity of stathmin in fibroblasts contributes to the stability of the microtubule network, while the abundance of stathmin in hematopoietic cells facilitates the disassembly of spindle microtubules and the disposal of midbodies. To test the latter possibility, we generated *Sept7* ^flox/flox^ MEFs that express stathmin via a doxycycline-regulatable promoter (Figure 5B). Indeed, stathmin overexpression (to the level of thymocytes) was sufficient to rescue the mitotic failure of *Sept7* ^−/−^ MEFs (Figure 5C, 5D) without changing other complex cellular properties as represented by cell mobility and adhesion measured in a scratch assay (Figure S11). We then asked whether stathmin overexpression also rescues multinucleation of the *Sept7* ^−/−^ MEFs. For this reason we co-transduced MEFs with pRBid–Cre and the doxycylin-inducible stathmin construct and DAPI-stained and counted mCherry-positive mono- and multinucleated cells after 5 days of cultivation in the presence or absence of doxycycline (Figure 5E and Figure S12). While the majority of control cells are multinucleated, overexpression of stathmin clearly shifted the MEFs to the mononucleated phenotype.

### Stathmin knockdown renders proliferation of Jurkat cells septin-dependent

Finally, we ask whether hematopoietic cells proliferating septin-independently require stathmin and whether stathmin-knockdown renders these cells sensitive to septin inactivation. To answer these questions we used the Jurkat human lymphocyte cell line, because manipulation of primary mouse hematopoietic cells in culture was not feasible. To inactivate septins in Jurkat cells, we applied the septin inhibitor forchlorfenuron (FCF) [31], which dampens septin dynamics and induces the assembly of abnormally large septin structures [32]. Stathmin knockdown by siRNA was performed and cells were further cultivated for 48 hours in the presence or absence of different concentrations of FCF (Figure 5F, 5G). siSTMN1 treatment efficiently reduced stathmin levels while the control siRNA did not (Figure 5F). Remarkably, while 50 µM FCF did not inhibit proliferation of Jurkat cells transfected with the control siRNA, siSTMN1-treated Jurkat cells displayed a clear proliferation defect at this concentration of FCF. At higher concentrations of FCF (100 µM) slight cytotoxic effects also reduced proliferation of the control, but the stronger reduction in the siSTMN1-treated cells remained. Taken together, we demonstrated that stathmin can rescue the proliferation block in SEPT7-deficient MEFs and that stathmin is necessary for proliferation of hematopoietic cells in the absence of functional septins. Thus, stathmin is a critical permissive factor whose abundance enables cells to proliferate without septins.

## Discussion

This study has revealed two distinct types of mammalian cytokinesis which vary by the requirement for SEPT7/septins. Consistent with previous studies [5], [16], [18] our findings indicate that cell division requires septins in two spatiotemporally distinct processes, first for the organization of the contractile ring and later for midbody abscission. The former became known early on due to the high prominence and its evolutionarily conservation from budding yeast to humans, while the latter had remained unknown due to its cell-type-dependence. Fibroblasts, typical adherent cells, divide in contact with other cells and/or connective tissue *in vivo* and extracellular matrices and artificial substrate *in vitro*. In contrast, amoeboid hematopoietic cells grow planktonically *in vivo* and divide individually in suspension. Our study confirm the role of septins in the recruitment of the microtubule cleaving machinery (multi-protein membrane associated abscission machinery probably including spastin for local microtubule destabilization) [7], [8] to the midbody for final microtubules scission. This system seems to be inactive in the absence of SEPT7 in fibroblasts, leading to midbody stabilization. In the hematopoietic system the abundance of stathmin leads to a passive rescue due to general microtubule destabilization and thus cytokinesis proceeds in a septin independent manner. The supplementation of stathmin is sufficient for fibroblasts to override the loss of SEPT7 and to complete cytokinesis. The abundant expression of stathmin in early embryo [33], [34] may account for the dispensability of septins up to midgestation. These data indicate that the synergy between septins and stathmin, among other microtubule-regulating proteins, is critical for completion of cytokinesis and midbody abscission. The entire process should depend not only on the quantitative balance of tubulin/stathmin/septin but also on the phosphorylation level of stathmin [29], [30]. Of note, β1-integrin-blocking antibodies can inhibit cytokinesis of adherent cells, but not their cytokinesis in suspension [35]. Given these and our findings, it is conceivable that non-adherent cells develop less cytoskeletal network than adherent cells, which should reduce the burden for midbody abscission. Conversely, myosin II-deficient *Dictyostelium* cells can complete cytokinesis on a substrate but not in suspension [36], indicating that microtubule is not a critical determinant in this case. A recent study with *Drosophila* revealed that the SEPT7 ortholog peanut (Pnut) and other septins are required for planar cell cytokinesis but dispensable for orthogonal cell division in the single-layered neuroepithelium of the dorsal thorax [37]. This finding supports our notion that SEPT7/septins play a context-dependent role in mammalian cytokinesis. Accordingly, SEPT7 is a promising target for the development of solid tumor-selective anti-proliferative therapy without damaging hematopoietic cells. Reciprocally, stathmin could be selectively targeted in hematopoietic malignancies and p53-compromized cancer [38], [39].

## Materials and Methods

### Generation of SEPT7 conditional knockout mice

Two independently developed *Sept7* floxed mice strains were used in this study, both targeting exon4 of mouse *Sept7* gene using similar targeting strategies. *Sept7* ^flox/flox^ mice (*Sept7^tm1Mgl^*) were generated as indicated in Figure 1A. Briefly, the targeting vector containing lox sites and FRT sites flanked neomycin cassette was linearized and electroporated in 129Ola ES-cells. Two positive clones (42A3 and 44A1) obtained by PCR screen were injected into blastocysts for the generation of chimeric mice. Agouti germ line pups were derived from the mating of chimeric male mice, obtained following the blastocyst injection of *Sept7* targeted ES-cell clone 44A1, with C57Bl/6 Flip females. The resulting sept7loxNeo mice were crossed with C57BL/6-(C3)-Tg(Pgk1-FLPo)10Sykr/J Flippase- expressing mice [40] to delete the neomycin cassette retaining the lox-P-flanked (floxed) exon 4 leading to *Sept7* ^lox^ mice. Subsequent Cre-recombinase expression will then catalyze exon 4-excision resulting in an additional frame-shift mutation downstream to this exon. For generation of Oocyte specific knockout animals, *Sept7* homozygous floxed mice were crossed with B6-*Zp3Cre^tmTgCre^* [41]. *Sept7^wt/flox^:Zp3Cre* mice were bred to generate *Sept7^wt/del^* mice. Lymphocyte-specific *Sept7* knockouts were generated by mating floxed animals with B6-*hCD2-iCre* mice [28]. In animal experiments age and sex matched, Cre-expressing *Sept7* wt and floxed mice were compared.

### DNA isolation and genotyping

Tail biopsies, cells and colonies were overnight digested at 53 °C in lysis buffer (50 mM Tris-Cl (pH 8.0), 100 mM EDTA, 100 mM NaCl and 1% SDS) containing proteinase-K (0.5 mg/mL). For tissue samples proteins were salted out with extra NaCl. DNA was precipitated with isopropanol, washed with 70% ethanol and dissolved in water. Genotyping PCR were performed with Hotstar Taq (Qiagen) with extra Mg^2+^ under standard conditions with annealing temperature at 53 °C. The primers used were- Sept7-p1 (5′- GGT ATA GGG GAC TTT GGG G-3′), Sept7-p2 (5′- CTT TGC ACA TAT GAC TAA GC -3′), Sept7-p3 (5′- GCT TCT TTT ATG TAA TCC AGG -3′), Cre-sense (5′- GAA CCT GAT GGA CAT GTT CAG G -3′), Cre-antisense (5′- AGT GCG TTC GAA CGC TAG AGC CTG T -3′), iCre-fwd (5′-AGA TGC CAG GAC ATC AGG AAC CTG- 3′), iCre-rev (5′-ATC AGC CAC ACC AGA CAC AGA GAT C- 3′), IL2-fwd (5′-CTA GGC CAC AGA ATT GAA AGA TCT- 3′), Il2-rev (5′-GTA GGT GGA AAT TCT AGC ATC ATC C- 3′), Myo-fwd (5′- TTA CGT CCA TCG TGG ACA GC -3′), Myo-rev (5′- TGG GCT GGG TGT TAG CCT TA -3′). Myogenin and IL2 gene fragments were amplified as controls for Cre and iCre genotyping respectively. PCR reactions were separated on 2% agarose gels and images acquired using INTAS Gel documentation system.

### Embryonic lethality analysis

*Sept7^wt/del^* mice were mated and plug checked for embryo analysis. Pregnant mice were sacrificed between embryonic day 6–7.0 or 10.5 days. The embryos were dissected out in cold PBS and cleaned up from extra-embryonic tissues. Whole embryos were overnight digested for DNA isolation and genotyping. Deviations from Mendelian ratios were calculated by Chi-squared test.

### Cell culture methods

*Sept7* floxed mouse embryonic fibroblasts were generated from E15 day embryos and maintained under standard conditions. *Sept7* floxed adult tail fibroblasts (TFs) were isolated from 6–8 weeks old mice tail tips. Minced tail tips were sequentially digested with collagenase and trypsin at 37°C and plated on collagen coated dishes in DMEM supplemented with 20% serum, non-essential amino acids and antibiotics. The cells were splitted 1∶4 and maintained in the same growth medium without coated dishes. To immortalize primary TFs, cells were co-transfected with pSV40Tag encoding simian virus 40 large T antigen and pREP8 plasmid (Invitrogen) in a 10∶1 mixture; colonies were selected with 2 mM histidinol (Sigma). Jurkat cells were maintained in RPMI-1640 medium supplemented with 15% serum, 1 mM pyruvate and antibiotics. Post electroporation cells were additionally supported by 2 ng/ml IL2.

### Antibodies and reagents

Antibody against SEPT7 was from IBL international (#JP18991), Rabbit anti-anillin antibodies were reported earlier (Watanabe *et al.*, 2010). Antibodies used for western blot analysis were SEPT2 (#11397-1-AP, Acris), SEPT9 (#10769-1-AP, Acris), SEPT6 (sc-20180, Santa Cruz Biotech), SEPT8 (sc-48937, Santa Cruz Biotech), EF2 (sc-13004-R, Santa Cruz Biotech), GAPDH (#MAB374, Millipore), GFP (sc-9996, Santa Cruz Biotech) and Stathmin (#3352, Cell Signaling Technology). Antibodies used for Immunofluorescence staining were rabbit anti SEPT7, SEPT2, SEPT6 [23], Ki67, phospho-Histone-H3, cleaved caspase-3 (Cell Signaling Technology), tubulin-α (T6199, Sigma and sc-31779, Santa Cruz Biotech), LAP2 (#611000, BD Transduction lab) and acetyl tubulin (T6793, Sigma). All alexa-dye labeled secondary antibodies, tetramethyl rhodamine-conjugated WGA (#W849) and Alexa fluor-647-conjugated phalloidin (#A22287) were from Invitrogen. DAPI for DNA staining was from Carl Roth (#6335.1). Polybrene (H9268), doxycycline (D9891), RNAse A (R4875) and propidium iodide (P4170) were from Sigma. Forchlorfenuron (FCF) was obtained from Santa Cruz Biotech. IL2 was from ImmunoTools. IL3, IL6 and SCF were from Peprotech.

### Virus transduction

Primary MEFs were transduced with commercially available adenoviral Cre particles (AxCANCre2, TaKaRa, Japan). Gammaretroviral particles (SF91-nlsCre and pRBid–Cre) were packaged as described previously [27]. Doxycycline inducible retroviral expression vector used for generating Stathmin-IRES-EGFP cell line was packaged as described previously [42]. Immortalized tail fibroblasts were seeded in 24 well plates (2.5×10^4^ cells/well) day before transduction. Plates with viral particles in the presence of polybrene (8 µg/mL) were spun at 1200× g for 1 h at 32°C. After overnight virus treatment, cells were washed, medium changed and processed as indicated. For *Sept7* deletion in primary lineage negative bone marrow progenitors, cells were trasduced by spinoculation with pRBid-Cre as described for tail fibroblasts. The transduction was repeated to achieve better transduction efficiency.

### Western immunoblotting

Cells were lysed directly in SDS gel loading dye and western blotting was performed as previously described using gradient SDS-PAGE gels [43].

### Immunofluorescence staining

Cells were grown on glass coverslips and fixed with 4% paraformaldehyde (PFA) in PBS. Fixation was performed for 2–5 min at room temperature (RT) followed by 20 min at 4°C. Cells were permeabilized with 0.25% Triton X-100–PBS for 30 min at RT. Blocking was done using 4% bovine serum albumin (BSA) for 1 h at 4°C. Primary antibodies were used at a 1∶50 to 1∶200 dilution in 1% BSA–PBS for 1–2 h. Secondary antibodies or Alexa Fluor 647-conjugated phalloidin/tetramethy rhodamine conjugated WGA was used at a 1∶500 dilution in 1% BSA–PBS. Imaging was performed using a Leica TCS SP2 confocal microscope with standard settings. Fluorescent intensities were quantified using Image J program (NIH- http://rsb.info.nih.gov/ij/).

### Flow cytometry analysis

For immunophenotyping analysis of hematopoietic cells, spleen, thymus and bone marrow cells were isolated, RBC lysed (Pharmlyse, BD Biosciences) and analyzed for surface staining with αCD3-FITC (Clone 17A2) [44], αB220-eFluor450 (Clone RA3-6B2, eBioscience), αCD4-PerCP (Clone RM4-5, Biolegend) and αCD8β-Cy5 (Clone RmCD8-2) [44]. Samples were analyzed using an LSRII (BD Biosciences).

For SEPT7, acetyl tubulin and propidium iodide staining, thymocytes/splenocytes were fixed with 3× by volume PFA (4%) at RT for 30 min. Washed and resuspended in PBS and absolute methanol was added to 90% concentration final with constant mixing. The methanol permeabilization was continued for 30 min on ice. After 2× PBS wash cells were resuspended in 4% BSA-PBS and blocked at 4°C for 30 min. Cells were stained with primary antibodies (1∶100 in 1%BSA-PBS) at RT for 30 min. After 1× PBS wash, samples were resuspended in secondary antibody dilution (anti rabbit Alexa fluor-488/anti mouse Alexa fluor-546 - 1∶500 diluted in 1% BSA-PBS) and incubated for additional 30 min before PBS wash and FACS analysis. For analysis of DNA content fixed cells were treated with nuclear stain solution (1× PBS, 100 µg/mL propidium iodide, 100 µg/mL RNAse-A) at RT for 15 min and analyzed by flow cytometry in Accuri- C6 flow cytometer.

### Time lapse-DIC microscopy

Cells were grown on 8 well chamber slides and time-lapse DIC images were acquired (1 per 10 min×16 h) using OLYMPUS FV1000 microscope fitted with 37°C/humid chamber.

### Methyl cellulose colony formation (CFC) assay

Bone marrow lineage negative cells were isolated by MACS separation (Miltenyl Biotech) and cells cultured for 2 days in the presence of IL3/SCF/IL6 medium. Cell were transduced on day 3 and 4 and left in suspension culture for another 4days. mCherry positive cells were sorted and seeded in 3 cm plates with methyl cellulose medium (IL3/IL6/SCF)(1000cells/ml/plate) as described previously [45]. Colonies were photographed, counted and genotyped after 2 weeks of growth.

### Peripheral blood analysis

Blood samples were collected in lithium-heparin tubes (BD Microtainer- LH tubes) and subjected to differential blood count and analysis with Vet ABC hematology analyzer (Scil animal care company GmbH, Viernheim, Germany).

### *In vitro* proliferation assay for splenocytes and fibroblasts

Spleens were asceptically isolated in RPMI medium (10% foetal calf serum, non-essential amino acids, antibiotics and 50 µM 2-mercapto ethanol) and splenocyte suspension obtained by passing through a 10 µm cell strainer. After RBC lysis cells/spleen were plated in a 6 cm plate and incubated at 37°C for 1 h to remove adherent cells. The suspension cells were collected and counted. 5–6×10^5^ cells/100 µl medium/well were seeded in 96 well plates in the presence or absence of 5 µg/ml concanavalin A and 10 ng/mL murine IL2. Cell proliferation was assayed using WST1 reagent (Roche Applied Sciences) as per manufacturer protocol. For measuring fibroblast proliferation, 500cells/100 µl/well were seeded in 96 well plates. Viable cells were quantified daily using WST1 reagent as per manufacturer protocol.

### Stathmin knockdown analysis in Jurkat cells

Jurkat cells were microporated with control siRNA (Allstars negative control siRNA- Qiagen) or siRNA against human Stathmin (Hs_STMN1_1: 5′-GCUGAGGUCUUGAAGCAGCTT-3′-Qiagen) using a Microporator MP-100 system. 200 picomoles of siRNA were used per one million cells microporated at 1400 V/20 msec/single pulse following the standard manufacturer's protocol. 24 h post transfection cells were counted and re-seeded at 3×10^5^/ml density in the presence or absence of FCF in v-bottom 96well plates (triplicate wells). After 48 h of treatment cells were collected and viable cell numbers quantified by flow cytometry in the presence of 2 µg/ml propidium iodide and 2 mM EDTA.

### Cell migration assay

Quantitative microplate scratch assays were performed with mitomycin-C treated fibroblasts as described previously [46]. Stathmin-IRES-EGFP transduced and sorted *Sept7* ^flox/flox^ cells were stained and seeded in 96 well plates in the presence or absence of 2 µg/ml doxycycline and scratches were made 24 h later after mitomycin pre-treatment. Infrared fluorescent images were acquired using a Li-COR odyssey scanner at 0 h, 6 h and 18 h. Migration indices were calculated and plotted.

### Ethics statement

All mice experiments were conducted according to German and international guidelines and were approved by the ethics committee of Hannover Medical School (MHH).

## Supporting Information

Figure S1 Down-regulation of anillin in SEPT7-depleted fibroblasts. Band intensities for SEPT7 and anillin blots presented in figure 1C were quantified and normalized to GAPDH. The data for two different floxed lines are presented as percentage of non-transduced control.
(PDF)

Figure S2 *Sept7* deletion does not induce enhanced cell death. Adenoviral Cre-transduced or control treated *Sept7^flox/flox^* primary MEFs were analyzed for apoptotic cells by cleaved caspase-3 staining. Cells with typical apoptotic morphology and cleaved caspase-3 staining (shown in set) were counted and plotted.
(PDF)

Figure S3 Microfilament and microtubule architecture in the SEPT7-deficient fibroblasts. **A,** Immortalized *Sept7^flox/flox^* fibroblasts transduced with retroviral Cre showing unaltered F-actin staining in the absence of SEPT7. **B,** SEPT7 knockout primary MEFs showing unaltered F-actin staining and **C,** enhanced microtubule acetylation. **D,** General microtubule architecture is unaffected in SEPT7-deficient immortalized fibroblasts as shown by α-tubulin staining. **E,** Intensity of acetyl tubulin (green)/SEPT7 (red) staining were quantified from individual cells using ‘Color histogram’ plugin of Image J program (n = 10). Representative images used for analysis are shown in the right panel with the quantified intensity values.
(PDF)

Figure S4 Dynamics of cell division in SEPT7-deficient fibroblasts. Time-lapse images were acquired for Cre-transduced *Sept7^flox/flox^* tail fibroblasts as described in methods, Figure 3A and supporting video S1. Total time taken for individual cells to complete cytokinesis was calculated. **A,** Sample time-lapse analysis showing a cell (indicated by red arrow) undergoing the complete process from cell detachment to complete abscission in 80 min. **B,** Similar analysis of all successful divisions in 39 distinct cells followed by time lapse. **C,** Classification of mitotic cells followed by time-lapse- including cells completing division (compiled from b) and cells failing to complete cell division.
(PDF)

Figure S5 Staining for LAP2 in unresolved midbody structures in *Sept7* KO fibroblasts. Upper and lower panel show two representative *Sept7* floxed tail fibroblast cells transduced with mCherry-Cre and stained with indicated antibodies. LAP2/Tubulin/DNA triple staining revealed the presence of unresolved midbody structures lacking chromosome bridges.
(PDF)

Figure S6 Bidirectional retroviral vector for expression of Cre and mCherry. **A,** Expression cassette and important features in the bidirectional Cre-mCherry expression vector used in the study. **B,** mCherry positive cells show efficient *Sept7*-deletion as shown by SEPT7 co staining in Cre-transduced cells. **C,** Representative images of mCherry positive hematopoietic cell colonies genotyped and enumerated in Figure 4.
(PDF)

Figure S7 Analysis of bone marrow in the Lymphocyte specific *Sept7* KO. **A,** *Sept7* genotyping showing partial deletion in CD2iCre mice bone marrow. Tail biopsy DNA is shown as a control tissue. **B,** Lymphocytes: T cells (CD3+) and B cells (B220+) in the bone marrow from CD2iCre mice (n = 2) were analyzed by surface-staining and flow cytometry.
(PDF)

Figure S8 Analysis of peripheral blood from lymphocyte specific *Sept*7 KO mice. Peripheral blood samples from *Sept7* ^flox/flox^::CD2-iCre mice (2×) and *Sept7* ^wt/wt^::CD2-iCre mice (3×) were analyzed using an ABC Vet Automated Blood counter (Scil animal care company GmbH, Viernheim, Germany).
(PDF)

Figure S9 Co-depletion of other septins in *Sept7* KO thymocytes. Similar to fibroblasts, *Sept7* deletion (CD2iCre) in thymocytes lead to depletion of SEPT2/6/9.
(PDF)

Figure S10 Analysis of tubulin acetylation in *Sept7* KO thymocytes. **A,** Thymocytes from *Sept7* ^flox/flox^::CD2-iCre mice (KO1 and KO2) and *Sept7* ^wt/wt^::CD2-iCre mice (WT1) were analyzed by indirect fluorescence staining and flow cytometry analysis for acetylated tubulin. Labeled Secondary antibody only staining is shown as control. **B,** As positive control for flow-cytometric detection of acetyl tubulin, similar staining was performed with control and taxol (2 µM for 2 h) treated Jurkat cells.
(PDF)

Figure S11 Effect of stathmin expression on fibroblast migration. *Sept7* floxed MEFs inducibly expressing stathmin were subjected to scratch wound healing assay. **A**, Representative fluorescent scans of wells showing scratch wound healing. **B,** Calculated migration index for 6 and 18 h wound healing (n = 19).
(PDF)

Figure S12 Analysis of multinucleation in stathmin expressing *Sept7* KO fibroblasts. *Sept7* floxed MEFs inducibly expressing stathmin were transduced with Rbid-Cre, maintained in the presence or absence of 2 µg/ml doxycycline and were fixed and stained with DAPI. The extent of multinucleation in mCherry-positive cells in the presence or absence of doxycycline- induced stathmin expression was quantified and is presented in Figure 5E.
(PDF)

Video S1 Stalled abscission and cytokinetic failure in *Sept7* KO fibroblasts. Sequence from the time-lapse differential interference contrast (DIC) microscopy of Cre-transduced *Sept7* floxed tail fibroblasts corresponding to Figure 3A. The cell attempting division (middle right) cannot resolve the intercellular bridge and does not complete cytokinesis after nuclear division. Even after 300 min the daughter cells do not separate and the cell becomes multi-nucleated.
(AVI)
