# Supplementary figures and images for: Genetic Deletion of SEPT7 Reveals a Cell Type-Specific Role of Septins in Microtubule Destabilization for the Completion of Cytokinesis
Source: PLoS Genet. 2014 Aug 14;10(8):e1004558. doi: 10.1371/journal.pgen.1004558 (PMC4133155; doi:10.1371/journal.pgen.1004558)

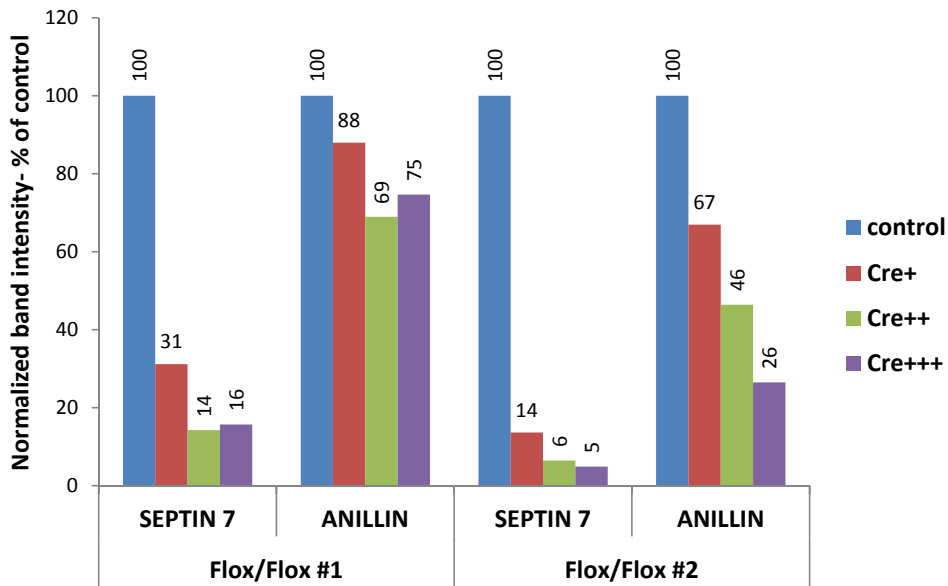

**Figure S1**

Supplement: Figure S1 — Down-regulation of anillin in SEPT7-depleted fibroblasts. Band intensities for SEPT7 and anillin blots presented in figure 1C were quantified and normalized to GAPDH. The data for two different floxed lines are presented as percentage of non-transduced control. (PDF) [file pgen.1004558.s001.pdf]

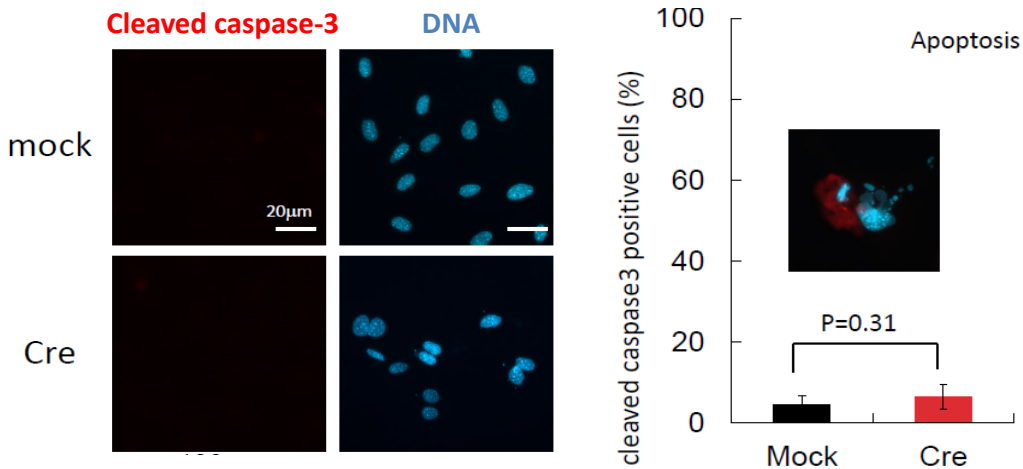

**Figure S2**

Supplement: Figure S2 — Sept7 deletion does not induce enhanced cell death. Adenoviral Cre-transduced or control treated Sept7flox/flox primary MEFs were analyzed for apoptotic cells by cleaved caspase-3 staining. Cells with typical apoptotic morphology and cleaved caspase-3 staining (shown in set) were counted and plotted. (PDF) [file pgen.1004558.s002.pdf]

**A**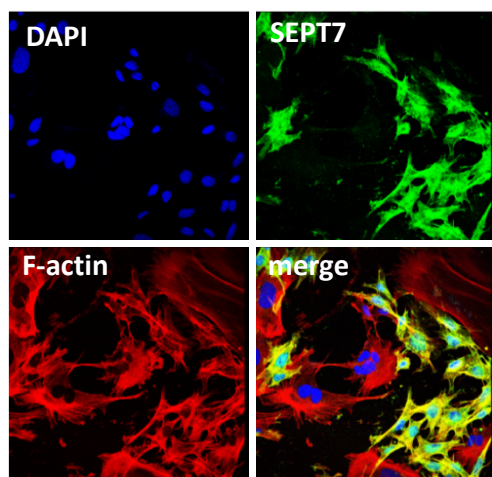**B**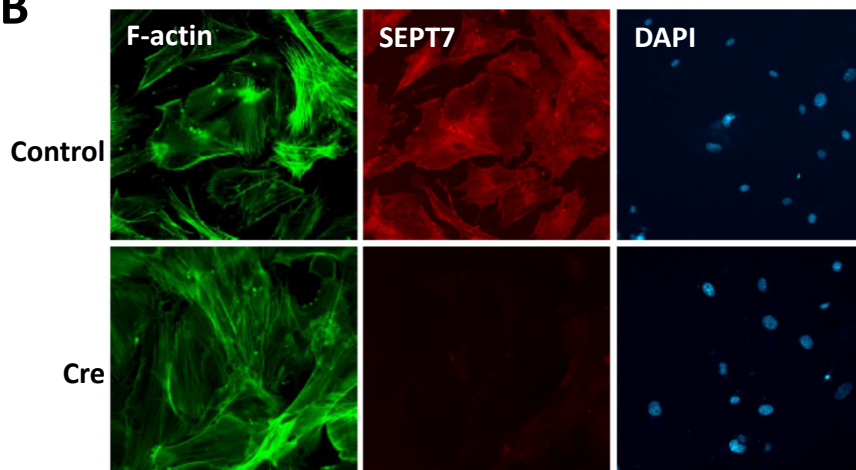**C**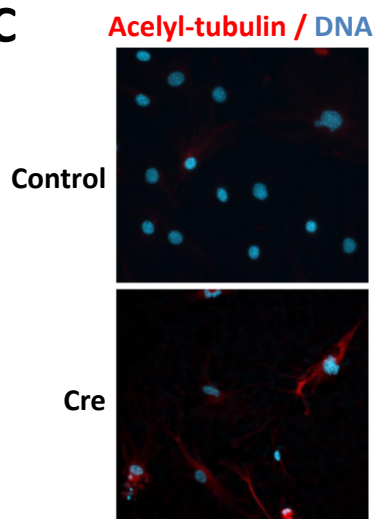**D**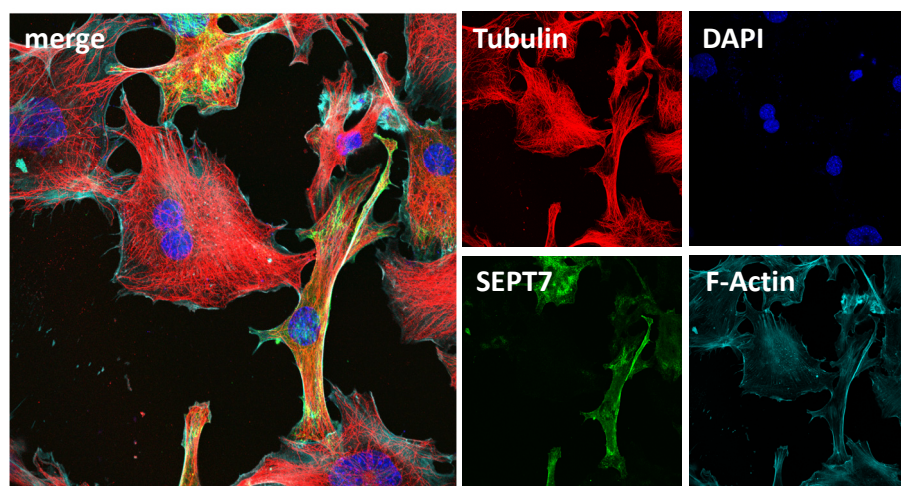**E**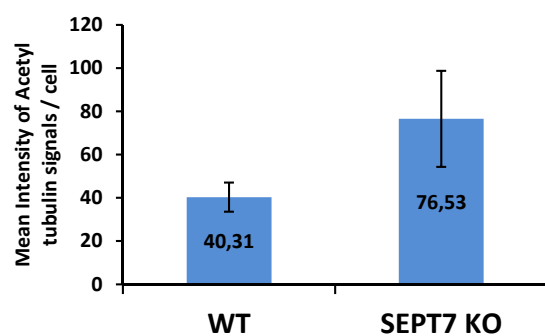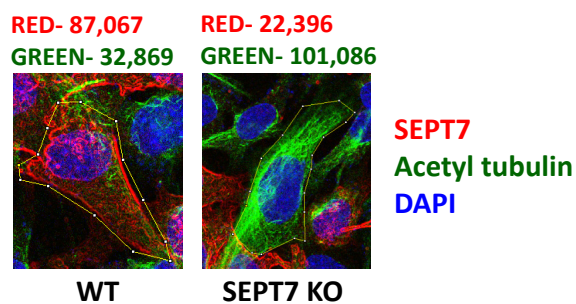**Figure S3**

Supplement: Figure S3 — Microfilament and microtubule architecture in the SEPT7-deficient fibroblasts. A, Immortalized Sept7flox/flox fibroblasts transduced with retroviral Cre showing unaltered F-actin staining in the absence of SEPT7. B, SEPT7 knockout primary MEFs showing unaltered F-actin staining and C, enhanced microtubule acetylation. D, General microtubule architecture is unaffected in SEPT7-deficient immortalized fibroblasts as shown by α-tubulin staining. E, Intensity of acetyl tubulin (green)/SEPT7 (red) staining were quantified from individual cells using ‘Color histogram’ plugin of Image J program (n = 10). Representative images used for analysis are shown in the right panel with the quantified intensity values. (PDF) [file pgen.1004558.s003.pdf]

**A**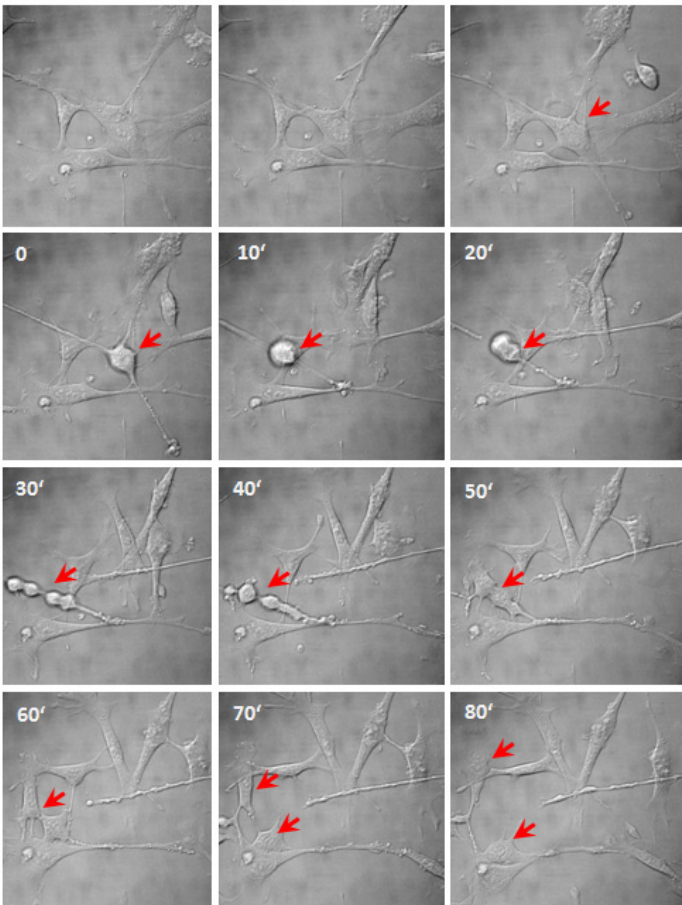**B**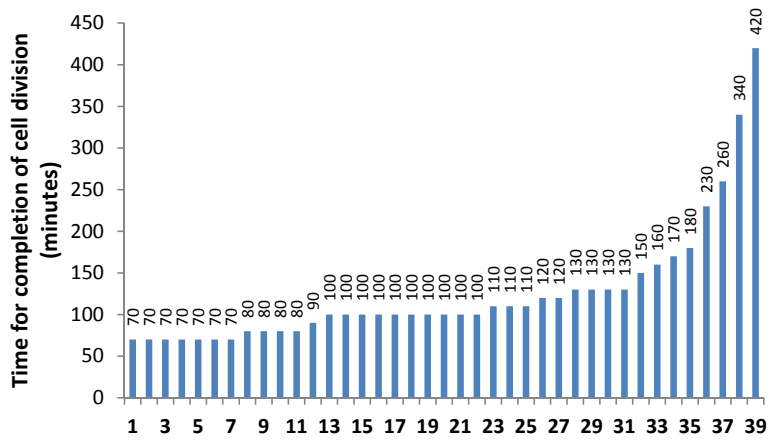**C**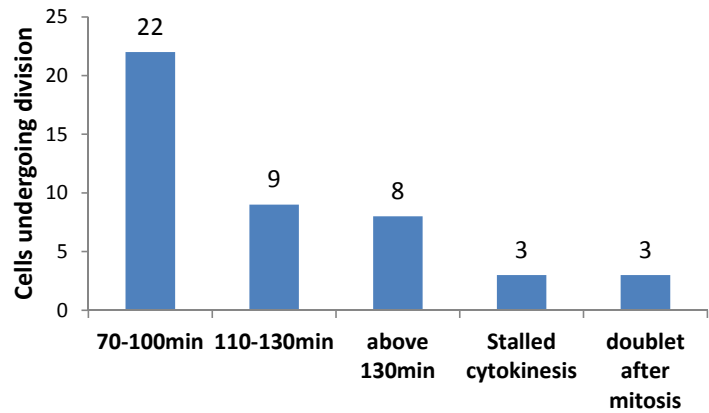

**Figure S4**

Supplement: Figure S4 — Dynamics of cell division in SEPT7-deficient fibroblasts. Time-lapse images were acquired for Cre-transduced Sept7flox/flox tail fibroblasts as described in methods, Figure 3A and supporting video S1. Total time taken for individual cells to complete cytokinesis was calculated. A, Sample time-lapse analysis showing a cell (indicated by red arrow) undergoing the complete process from cell detachment to complete abscission in 80 min. B, Similar analysis of all successful divisions in 39 distinct cells followed by time lapse. C, Classification of mitotic cells followed by time-lapse- including cells completing division (compiled from b) and cells failing to complete cell division. (PDF) [file pgen.1004558.s004.pdf]

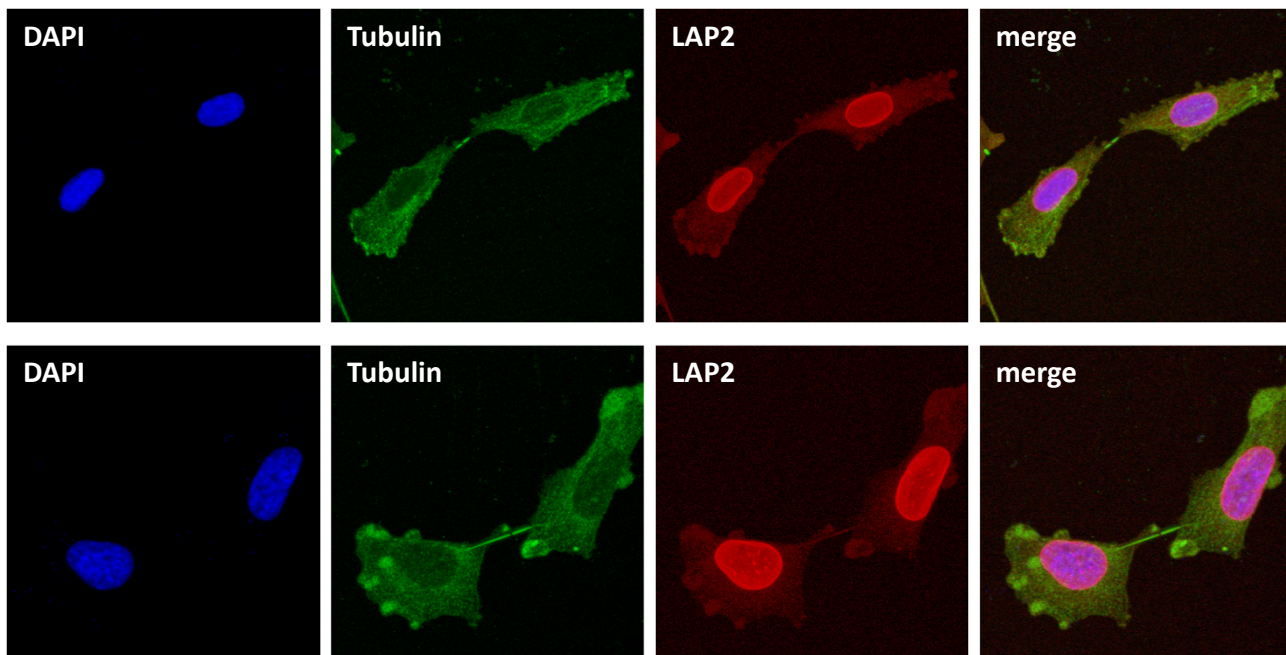

**Figure S5**

Supplement: Figure S5 — Staining for LAP2 in unresolved midbody structures in Sept7 KO fibroblasts. Upper and lower panel show two representative Sept7 floxed tail fibroblast cells transduced with mCherry-Cre and stained with indicated antibodies. LAP2/Tubulin/DNA triple staining revealed the presence of unresolved midbody structures lacking chromosome bridges. (PDF) [file pgen.1004558.s005.pdf]

**A**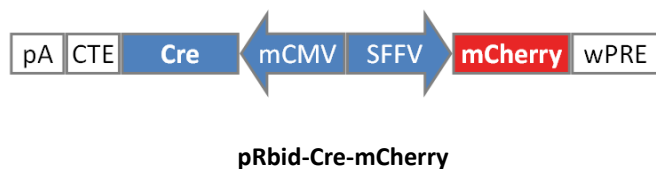**B**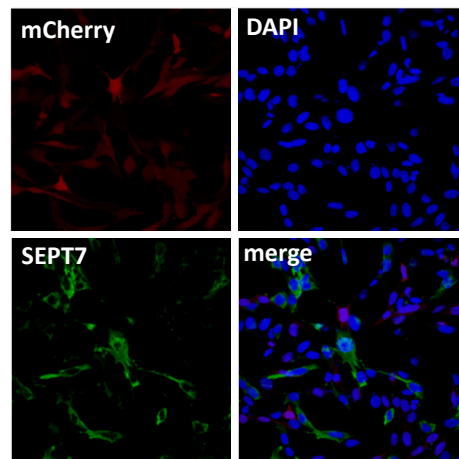**C**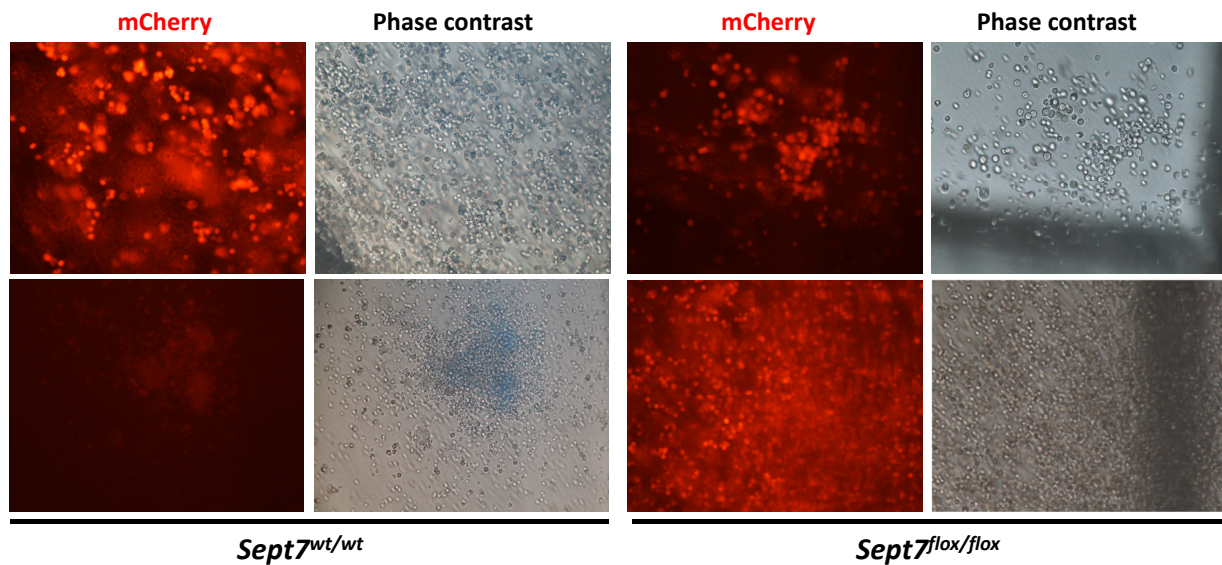**Figure S6**

Supplement: Figure S6 — Bidirectional retroviral vector for expression of Cre and mCherry. A, Expression cassette and important features in the bidirectional Cre-mCherry expression vector used in the study. B, mCherry positive cells show efficient Sept7-deletion as shown by SEPT7 co staining in Cre-transduced cells. C, Representative images of mCherry positive hematopoietic cell colonies genotyped and enumerated in Figure 4. (PDF) [file pgen.1004558.s006.pdf]

**A**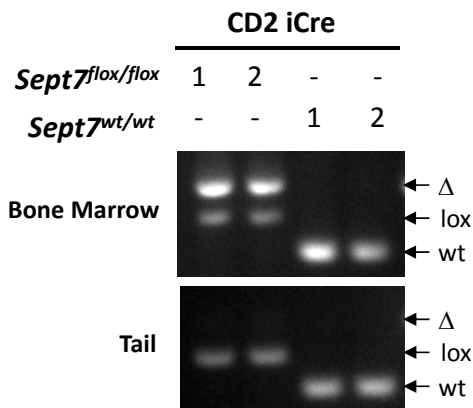**B**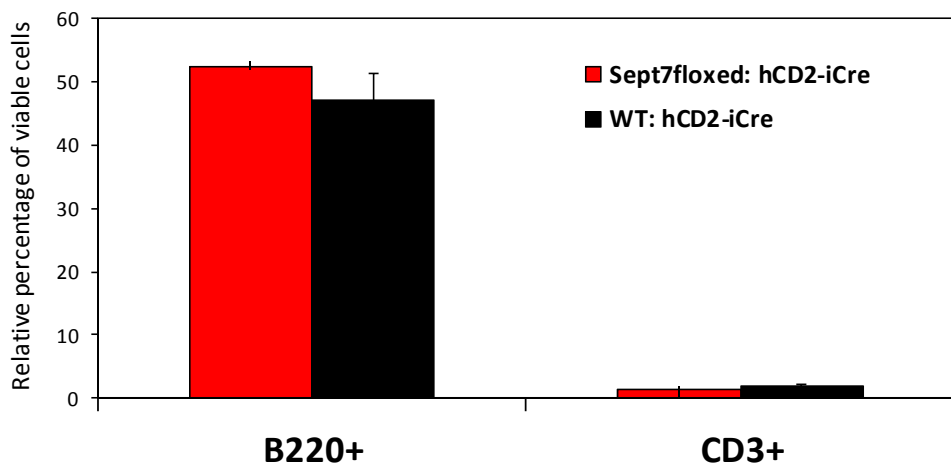**Figure S7**

Supplement: Figure S7 — Analysis of bone marrow in the Lymphocyte specific Sept7 KO. A, Sept7 genotyping showing partial deletion in CD2iCre mice bone marrow. Tail biopsy DNA is shown as a control tissue. B, Lymphocytes: T cells (CD3+) and B cells (B220+) in the bone marrow from CD2iCre mice (n = 2) were analyzed by surface-staining and flow cytometry. (PDF) [file pgen.1004558.s007.pdf]

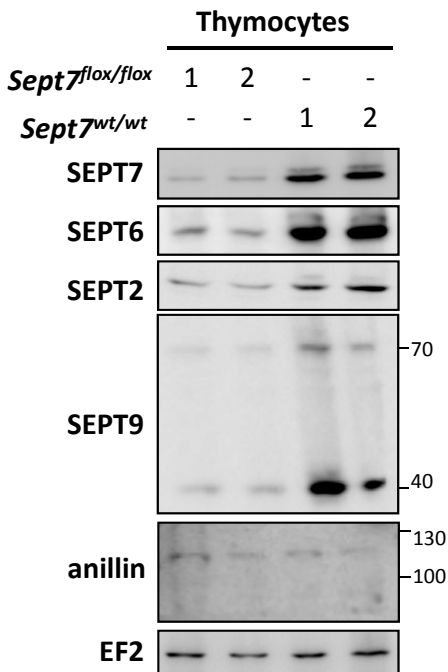

**Figure S9**

Supplement: Figure S9 — Co-depletion of other septins in Sept7 KO thymocytes. Similar to fibroblasts, Sept7 deletion (CD2iCre) in thymocytes lead to depletion of SEPT2/6/9. (PDF) [file pgen.1004558.s009.pdf]

**A**

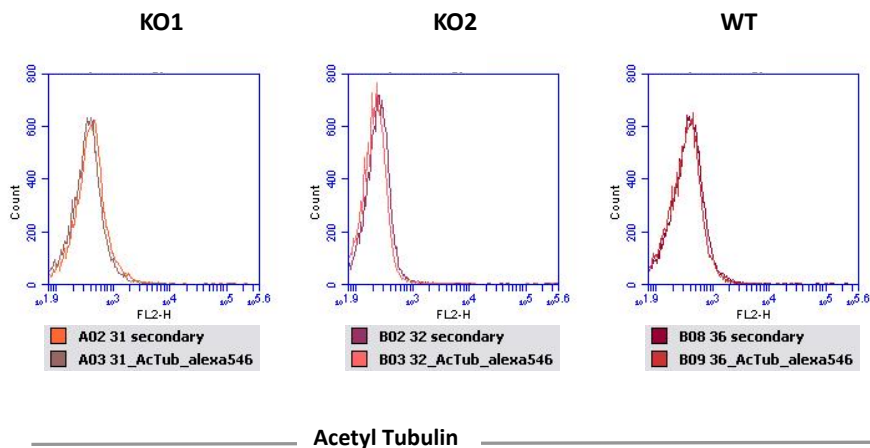

**B**

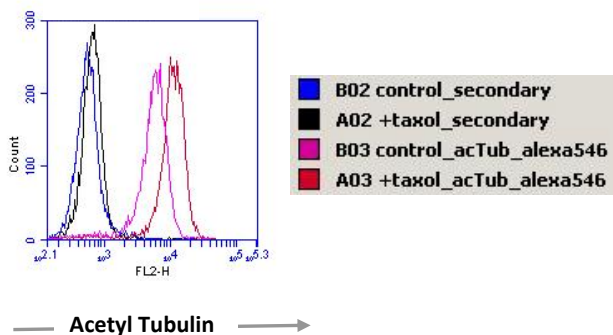

**Figure S10**

Supplement: Figure S10 — Analysis of tubulin acetylation in Sept7 KO thymocytes. A, Thymocytes from Sept7 flox/flox::CD2-iCre mice (KO1 and KO2) and Sept7 wt/wt::CD2-iCre mice (WT1) were analyzed by indirect fluorescence staining and flow cytometry analysis for acetylated tubulin. Labeled Secondary antibody only staining is shown as control. B, As positive control for flow-cytometric detection of acetyl tubulin, similar staining was performed with control and taxol (2 µM for 2 h) treated Jurkat cells. (PDF) [file pgen.1004558.s010.pdf]

**A**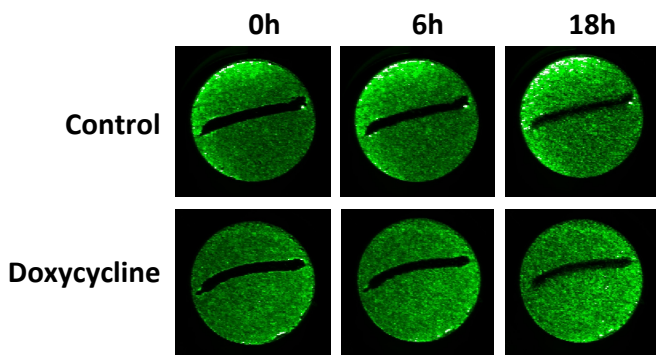**B**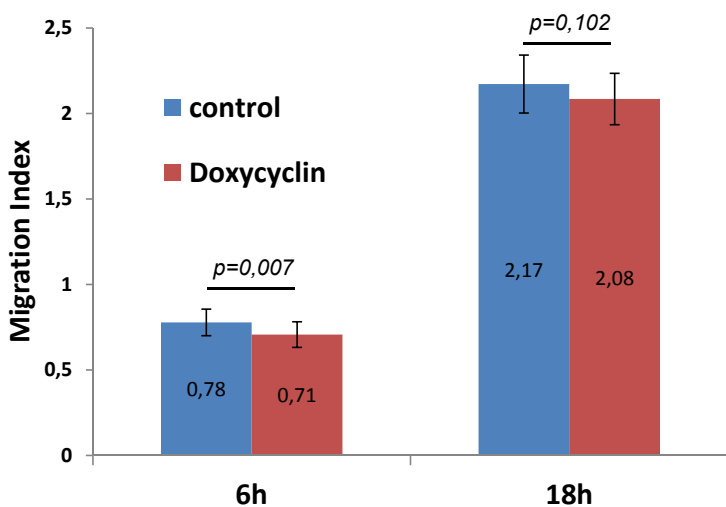**Figure S11**

Supplement: Figure S11 — Effect of stathmin expression on fibroblast migration. Sept7 floxed MEFs inducibly expressing stathmin were subjected to scratch wound healing assay. A, Representative fluorescent scans of wells showing scratch wound healing. B, Calculated migration index for 6 and 18 h wound healing (n = 19). (PDF) [file pgen.1004558.s011.pdf]

*Sept7*<sup>flox/flox</sup> MEF + pSERS-Stmn1-IRES-GFP + pRbid-Cre

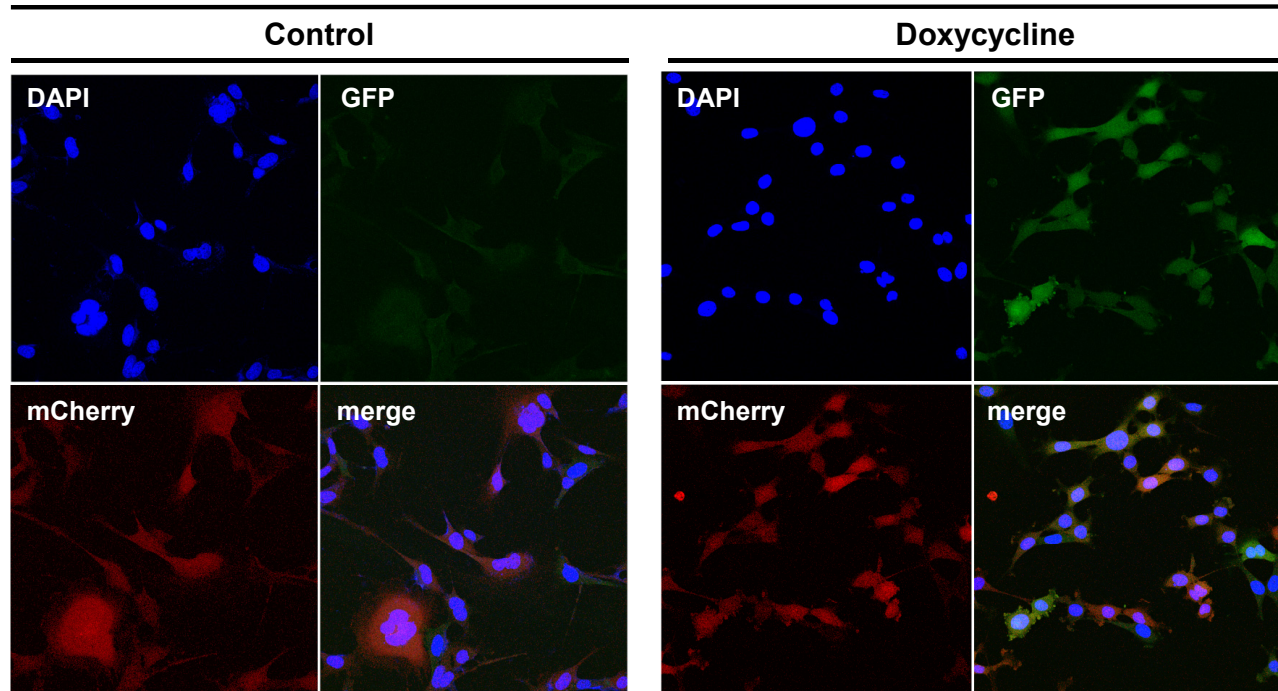

Figure S12

Supplement: Figure S12 — Analysis of multinucleation in stathmin expressing Sept7 KO fibroblasts. Sept7 floxed MEFs inducibly expressing stathmin were transduced with Rbid-Cre, maintained in the presence or absence of 2 µg/ml doxycycline and were fixed and stained with DAPI. The extent of multinucleation in mCherry-positive cells in the presence or absence of doxycycline- induced stathmin expression was quantified and is presented in Figure 5E. (PDF) [file pgen.1004558.s012.pdf]
