# Supplementary material for: Genetic Deletion of SEPT7 Reveals a Cell Type-Specific Role of Septins in Microtubule Destabilization for the Completion of Cytokinesis
Source: PLoS Genet. 2014 Aug 14;10(8):e1004558. doi: 10.1371/journal.pgen.1004558 (PMC4133155; doi:10.1371/journal.pgen.1004558)
Supplement: Figure S8 — Analysis of peripheral blood from lymphocyte specific Sept7 KO mice. Peripheral blood samples from Sept7 flox/flox::CD2-iCre mice (2×) and Sept7 wt/wt::CD2-iCre mice (3×) were analyzed using an ABC Vet Automated Blood counter (Scil animal care company GmbH, Viernheim, Germany). (PDF) [file pgen.1004558.s008.pdf]

	Leucocytes (x 1000 cells/µL)	Erythrocytes (x 1000000 cells/µL)	Platelets (x 1000 cells/µL)	hemoglobin (g/dL)	Hematocrit (%)
Sept7$^{wt/wt}$	7,83+/- 1,12	11,53 +/- 0,55	139,7 +/- 12	16,7 +/- 0,8	60,4+/- 3,07
Sept7$^{flox/flox}$	6,205 +/- 1,49	9,07 +/- 1,03	337 +/- 73	14,62 +/- 0,98	49,6 +/- 4,4

	Lymphocytes (x 1000 cells/µL)	Monocytes (cells/µL)	Granulocytes (x 1000 cells/µL)	Eosinophils (cells/µL)
Sept7$^{wt/wt}$	5,8 +/- 1,47	333 +/- 57	1,73 +/- 0,40	433 +/- 58
Sept7$^{flox/flox}$	4,05 +/- 0,21	300 +/- 0	1,85 +/- 0,21	600 +/- 141

Figure S8
